# The care of older cancer patients in the United Kingdom

**DOI:** 10.3332/ecancer.2020.1101

**Published:** 2020-09-15

**Authors:** Fabio Gomes, Anna Lewis, Rob Morris, Ruth Parks, Tania Kalsi, Gordana Babic-Illamn, Mark Baxter, Kirsty Colquhoun, Lisa Rodgers, Eleanor Smith, Alastair Greystoke, Neil Bayman, Anthea Cree, Cassandra Ng, Nicola de Liguori Carino, Simone Basile, John Moore, Zoe Merchant, Daniel Swinson, Anita Parbhoo, Rachel Jones, Eleri Davies, Sarah J Danson, Robin Young, Jenna Morgan, Lynda Wyld, Pippa G Corrie, Gary J Doherty, Kyle Crawford, Juliet Wright, Malcolm Reed, Fiammetta Ugolini, Michael Lind, Kwok-Leung Cheung, Danielle Harari, Richard Simcock

**Affiliations:** 1 The Christie NHS Foundation Trust, Manchester M20 4BX, UK; 2 Nottingham University Hospitals NHS Trust, Nottingham NG5 1PB, UK; 3 School of Medicine, University of Nottingham, Nottingham NG7 2UH, UK; 4 Guy’s and St. Thomas’ NHS Foundation Trust, London SE1 9RS, UK; 5 King’s College London, London SE5 9RS, UK; 6 Ninewells Hospital, NHS Tayside, Dundee DD2 1SG, UK; 7 Beatson West of Scotland Cancer Centre, NHS Greater Glasgow and Clyde, Glasgow G12 0YN, UK; 8 The Newcastle upon Tyne Hospitals NHS Foundation Trust, Newcastle upon Tyne NE7 7DN, UK; 9 Wythenshawe Hospital, Manchester University NHS Foundation Trust, Manchester M23 9LT, UK; 10 Royal Manchester Infirmary, Manchester University NHS Foundation Trust, Manchester M13 9WL, UK; 11 Greater Manchester Cancer, Manchester M20 4BX, UK; 12 Leeds Teaching Hospitals NHS Trust, Leeds LS1 3EX, UK; 13 South West Wales Cancer Centre, Swansea Bay University Health Board, Swansea SA2 8QA, UK; 14 University Hospital of Llandough, Cardiff and Vale University Health Board, Cardiff CF64 2XX, UK; 15 Department of Oncology and Metabolism, The Medical School, University of Sheffield, Sheffield S10 2RX, UK; 16 Cambridge University Hospitals NHS Foundation Trust, Cambridge CB2 0QQ, UK; 17 Belfast Health and Social Care Trust, Belfast BT13 1FD, UK; 18 Brighton and Sussex Medical School, University of Brighton, Brighton BN1 9PX, UK; 19 Sussex Cancer Centre, Brighton and Sussex University Hospitals NHS Trust, Sussex BN2 5BD, UK; 20 Queen’s Centre for Oncology and Haematology, Hull University Teaching Hospitals NHS Trust, Hull HU16 5JQ, UK; a Co-senior authorship

**Keywords:** geriatric oncology, older patients, cancer, United Kingdom

## Abstract

The ageing population poses new challenges globally. Cancer care for older patients is one of these challenges, and it has a significant impact on societies. In the United Kingdom (UK), as the number of older cancer patients increases, the management of this group has become part of daily practice for most oncology teams in every geographical area. Older cancer patients are at a higher risk of both under- and over-treatment. Therefore, the assessment of a patient’s biological age and effective organ functional reserve becomes paramount. This may then guide treatment decisions by better estimating a prognosis and the risk-to-benefit ratio of a given therapy to anticipate and mitigate against potential toxicities/difficulties. Moreover, older cancer patients are often affected by geriatric syndromes and other issues that impact their overall health, function and quality of life. Comprehensive geriatric assessments offer an opportunity to identify and address health problems which may then optimise one’s fitness and well-being. Whilst it is widely accepted that older cancer patients may benefit from such an approach, resources are often scarce, and access to dedicated services and research remains limited to specific centres across the UK. The aim of this project is to map the current services and projects in the UK to learn from each other and shape the future direction of care of older patients with cancer.

## Introduction

Cancer is mostly a disease of older adults with about 60% of cases diagnosed in those aged 65 years and above [1]. In the United Kingdom (UK), the number of older adults living with a cancer diagnosis in 2010 was 1.3 million, but this is rapidly increasing and is estimated to reach 4.1 million by 2040 [2]. Older cancer patients can have wider health needs and are the majority; in fact, they represent everyday practice for oncologists across multiple cancer sites. The growing number of older cancer patients increases the demand on the National Health Service (NHS). Ageing is associated with increasing frailty which, in turn, may increase the complexity of care. Importantly, UK law is clear that age discrimination must not occur (Equality Act 2010 Order 2012), and therefore, the NHS cannot (and should not) provide inferior services solely because of a patient’s age. In the context of these important epidemiological and sociological drivers, there has been a significant effort in recent years across different sectors in the UK to improve care and research for older cancer patients.

## National Health Service (NHS)

In 2018, the NHS (England, Wales and Scotland) marked its 70th anniversary, and the NHS England Long Term Plan was developed [3]. This plan identified improving care for all older people living with frailty (such as cancer patients) as a priority. It includes enhancing health within care homes and also strengthening community teams to be more proactive and to support patients at home during a crisis. Specifically for cancer patients, one key aim has been to deliver a comprehensive model for personalised care with the assessment of needs and further integration of Clinical Nurse Specialists and other support workers, alongside increased community-based support. It is, therefore, hoped that the care for frail cancer patients, who are mostly older, should have a significant boost in resources in the coming years. In this context, and as a part of the NHS England RightCare Pathways, the NHS RightCare Frailty Toolkit was published in 2019 [4]. This resulted from a collaboration with National Clinical Director for Older People at NHS England, the charity Age UK, the NHS improvement programme Getting It Right First Time and the National Institute for Health and Care Excellence. This toolkit provides expert guidance on how to commission and provide the best system-wide care for people living with frailty. In essence, it helps us to understand the priorities in frailty identification and care, provides a benchmark and identifies key actions for improvement.

## Specialised Clinical Frailty Network (SCFN)

Frailty is a state of increased vulnerability to poor resolution of homoeostasis after a stressor event, which increases the risk of adverse outcomes. Moreover, frailty is associated with ageing; hence, in 2018, the NHS Elect was commissioned to deliver the SCFN in England. This is a clinically led quality improvement collaborative working with specialised teams across different medical areas to explore how frailty assessment and management can best be integrated. This network was launched following the success of the Acute Frailty Network developing front door frailty services in secondary care. A key element is the integration of the Rockwood clinical frailty scale (CFS) in routine clinical assessments [5]. This nine-level scale provides a quick and simple method to identify frailty and is based on how active and independent patients are on their daily life activities. The first wave of specialised services started in late 2018 and included five cancer services across England, focusing mainly on patients with lung cancer being considered for chemotherapy. More recently, in late 2019, the third wave was launched and expanded the scope to cancer surgery services with a focus on gynaecological cancer. Following on from the collaborative work developed within the first two waves, the Specialised Clinical Frailty Toolkit was launched [6]. This toolkit is not exclusive to oncology and is targeted at anyone working with older people with frailty and involved in the design and delivery of specialised services. The aim is to provide guidance on how to develop the most appropriate pathways for frail patients within specialised services to optimise their outcomes.

## Cancer charities

In 2015, the cancer charity Macmillan convened a multidisciplinary Expert Reference Group (ERG) in cancer in the older person. This group scoped current assessment methods employed across the UK in cancer services and used this along with a nominal consensus approach to come to an expert consensus on the optimal geriatric oncology assessment tool for use in the UK [7]. The ERG also produced a consultation document [8] highlighting the need to educate the NHS workforce in issues pertinent to older people. Mutual education was recommended with geriatric specialists being upskilled in oncological issues and vice versa. To facilitate this, the ERG developed educational sessions and material for primary care teams and other healthcare professionals, and it also coordinated study days with the British Geriatric Society. Moreover, whilst being involved in the international Delphic process to establish the core curriculum in Geriatric Oncology, it promoted changes in the training curricula for medical and clinical oncologists in the UK. Beyond promoting education, the Macmillan ERG played a role in the development of specific recommendations in the NHS England Cancer Strategy plan for 2015–2020. These include piloting a comprehensive care programme for older people with cancer [assessment of holistic needs and comprehensive geriatric assessment (CGA)] and to develop dedicated research protocols [9].

Cancer Research UK is the largest cancer charity in the UK, and whilst it has no funding stream dedicated to geriatric oncology, it produced a report in 2018 on how to prepare for the increasing number of older patients [10]. This report echoed calls to assess frailty, implement CGA and introduce new processes in multidisciplinary team meetings. The report also asked for policy changes to NHS waiting time targets, arguing that, in older patients, the time spent before treatment in assessing and optimising fitness was valuable, yet potentially discouraged by these targets which mandate a rapid start of treatment.

## National Cancer Research Institute (NCRI)

Research is key, and the NCRI has been committed, through its working groups, to support research that considers the distinct issues of older people. The NCRI conducted a scoping exercise for priorities in living with and beyond cancer. Priority number 3 focuses on how to better coordinate care for people with complex needs living with and beyond cancer [11]. Moreover, the NCRI developed an important workshop highlighting these issues and suggesting ways to optimise the research design for older cancer patients [12, 13].

## Professional societies

The British Geriatrics Society (BGS) was founded in the UK in 1947 and is a multidisciplinary society that welcomes everyone specialising in the healthcare of older people. In 2015, the BGS Special Interest Group in Geriatric Oncology was founded. It is open to any healthcare professionals involved in the care of older cancer patients and aims to promote education, training and research in the field. Its long-term aims include an e-learning package for geriatricians, sharing examples of good practice and providing practical advice on how to develop business cases.

On the global stage, the UK has been actively involved within the International Society of Geriatric Oncology (SIOG), which was founded in 2000 and is growing rapidly. This multidisciplinary group’s sole purpose is to promote geriatric oncology and optimise the treatment of older adults with cancer. SIOG promotes efforts in three strategic directions: education, clinical practice and research. Its original 10 priorities’ proposal from 2011 is currently being revised, and publication is expected soon, which is an important initiative to advocate for better care and research in this field [14]. Finally, SIOG’s dedicated Journal of Geriatric Oncology has disseminated many guidelines and practice to change the research outcomes worldwide. The UK is continuously represented at SIOG through its national representative and also with members at the governance level.

## National audits in geriatric oncology

National audits are a good opportunity to understand practice and outcomes across the UK. The National Audit of Breast Cancer in Older Patients (NABCOP) is a comprehensive and on-going study of the pathways of care and outcomes for women diagnosed with breast cancer over the age of 70 (with comparative data obtained from a cohort of women aged 50–69 years). The audit has now produced a third annual report which has highlighted significant disparities in care [15]. The NABCOP has been successful in implementing the Rockwood CFS assessment as a field within the National Cancer Data Registry, making it the first disease site to mandate the collection of these data through a national dataset.

## Geriatric oncology services and projects across the UK

### England

**Figure 1.**
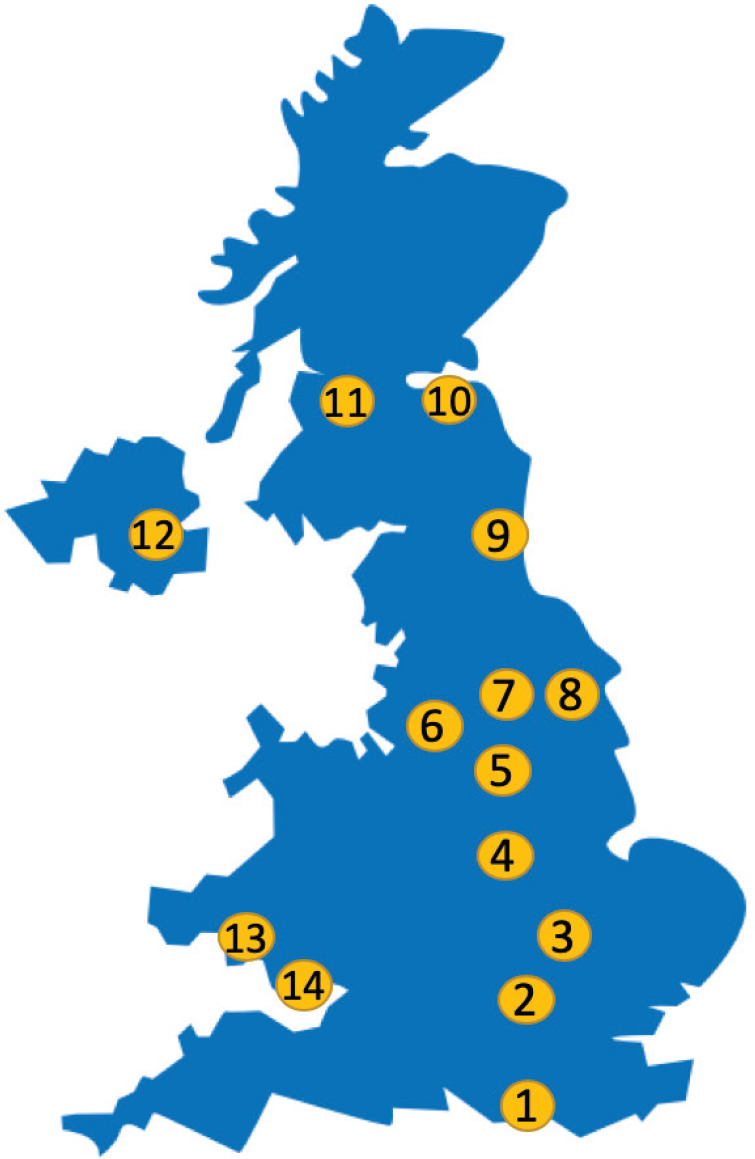
Geographic representation of key services and projects. 1—Brighton (Sussex Cancer Centre—Brighton and Sussex University Hospitals NHS Trust). 2—London (Guy’s and St. Thomas’ NHS Foundation Trust, University College London Hospitals NHS Foundation Trust). 3—Cambridge (Cambridge University Hospitals NHS Foundation Trust). 4—Nottingham (Nottingham University Hospitals NHS Trust). 5—Sheffield (Sheffield Teaching Hospitals NHS Foundation Trust). 6—Manchester (The Christie NHS Foundation Trust; Manchester University NHS Foundation Trust). 7—Leeds (Leeds Teaching Hospitals NHS Trust). 8—Hull (Queen’s Centre for Oncology and Haematology—Hull University Teaching Hospitals NHS Trust). 9—Newcastle upon Tyne (Newcastle upon Tyne Hospitals NHS Foundation Trust). 10—Dundee (Ninewells Hospital—NHS Tayside). 11—Glasgow (Beatson West of Scotland Cancer Centre). 12—Belfast (Belfast Health and Social Care Trust—NHS Greater Glasgow and Clyde). 13—Swansea (South West Wales Cancer Centre, Swansea Bay University Health Board). 14—Cardiff (Cardiff and Vale University Health Board).

The NHS England is comprised of seven regional teams according to geographic areas which serve as a population of approximately 56 million people (Figure 1). Cancer care is coordinated at a more local level through 20 cancer alliances which bring together key organisations in their areas.

#### Brighton

The Brighton and Sussex University Hospitals Trust developed a joint geriatric breast surgery clinic which is staffed by a surgeon and a geriatrician. This runs in parallel with a specialist oncology clinic and with support from breast cancer nurse specialists. Patients are referred at the start of their pathway if they are potentially unfit for, or they decline, the standard treatment for breast cancer. There are no specific age-based referral criteria. Patients are jointly assessed by the breast surgeon and geriatrician, who undertake an abbreviated CGA focussed on the management of comorbidities, functional status, polypharmacy, cognitive function, mood and social support. Following this joint assessment, the clinicians formulate a management plan with options that are discussed with the patient in a shared decision-making approach. For the patients planned for surgery, an anaesthetic review is also arranged.

In the period between April 2015 and March 2020, a total of 182 breast cancer patients were seen. First appointments averaged 45 minutes to include an abbreviated CGA, evaluation of breast cancer and discussion of diagnosis and management plan. The mean age was 82 years (range 69–99 years). 57% of patients had subsequent assessments/reviews (between 1 and 6 times), which averaged 25 minutes duration. Regarding their outcomes, 13% of patients were referred for surgery, and in 35%, the medical management was changed, typically for treatment optimisation, further investigations or referral to other specialists [16].

Future plans for the clinic include providing diagnostic clinics for patients identified as frail and for the joint care of suitable patients with advanced disease.

#### Cambridge

Cambridge University Hospitals NHS Foundation Trust (CUH) addressed the problem that ECOG and Karnofsky performance status scales, which are routinely used, are problematic in assessing older cancer patients’ pre-treatment. In 2016, they explored how the Rockwood CFS, which was developed in the acute/community setting, would perform in routine outpatient cancer clinics. A total of 114 cancer patients were assessed using these three clinical tools. The CFS was the best tool at differentiating patients below and above 70 years (*p* = 0.0056) followed by the Karnofsky (*p* = 0.02), whereas the ECOG performance status found no differences [17].

In 2018, the CUH lung cancer team joined the SCFN led by NHS Elect in England as one of the five pilot oncology teams. The team implemented a questionnaire incorporating the CFS and covering the different domains of CGA. This was divided into two parts, the first being patient-led and the second healthcare professional-led. It was targeted at all new advanced lung cancer patients being considered for systemic anticancer treatment with an ECOG performance status of 2+. The Rockwood CFS was used as a complementary measure but not to determine who might benefit from CGA. Ultimately, the team aimed at mapping frailty and the domains affected to better inform treatment decisions. This work further supported the use of CFS which provided more granularity compared with the conventional ECOG performance status. Moreover, the team reported that the information provided by these additional assessments helped to tailor treatment decisions, medicine discontinuation and appropriate referrals to therapists. It also highlighted a significant burden of physical and psychological factors contributing to frailty.

#### Hull

The Queen’s Centre for Oncology and Haematology in conjunction with Transform group from the Hull York medical school (funded by Yorkshire Cancer Research) is developing a programme of research in tandem with a service development for older cancer patients. Their initial research was to look at the feasibility of CGA in an oncology outpatient setting [18]. At present, they are in the process of developing an oncogeriatric service for patients with established or suspected cancer before they are discussed at multidisciplinary meetings so that decision about cancer care can be better made. Ultimately, this will involve collecting the data by smartphone or tablet apps.

The team is also developing a programme of tailored nutrition and exercise intervention for those over 70-year old with lung cancer (Can-Benefit) to improve symptoms and fitness for systemic cancer therapy. Finally, the team is also planning to address the barriers to clinical trial entry for these older cancer patients with a mixture of systematic reviews and stakeholder involvement.

#### Leeds

The Clinical Trial Unit in Leeds coordinated the recently published GO2 trial [19]. This is a multicentre phase 3 trial which compared three different doses of oxaliplatin and capecitabine in 514 frail/elderly patients with advanced gastroesophageal cancer and a definite indication for chemotherapy. This study recruited patients between 2014 and 2017 across 61 UK centres. In this trial, a comprehensive health assessment was performed before randomisation, and the study included a novel outcome measure, i.e., overall treatment utility (OTU). OTU is a composite endpoint derived from both clinical- and patient-reported outcomes and is scored as good, intermediate or poor. A good OTU is achieved if: 1) there is an absence of disease progression (clinical and radiological) and 2) there is no significant toxicity and good patient acceptability.

The presence of one adverse outcome results in an intermediate OTU and both a poor OTU. This population had a median age of 76 years, and 58% were scored as being very frail. The trial concluded that the lowest dose of chemotherapy achieved the highest rates of good OTU. An initial analysis found that paradoxically, the least frail and better performance status patients gained most from reducing the dose of chemotherapy, whereas age was not associated with worse outcomes. This trial confirmed that it is feasible to recruit frail/elderly patients into clinical trials. Moreover, it questions the long-held oncology paradigm that systemic anticancer therapy dosing should be based on the maximum tolerated doses. Future trials are needed to identify minimal effective dosing for the elderly/frail patients in other cancer sites.

#### London

At Guy’s and St. Thomas’ NHS Foundation Trust, the Geriatric Oncology Liaison Development (GOLD) service was a pioneer in the UK. It was established with a clear aim: to facilitate equity for older people’s cancer treatment decision-making, based on fitness for treatment and patient choice rather than chronological age. An initial screening questionnaire identifies medical, functional and/or psychosocial problems [20]. These are then addressed in a multidisciplinary outpatient clinic with geriatric-trained doctors and nurses, and occupational therapy and physiotherapy were needed to deliver an optimisation plan for older patients undergoing cancer treatment. In the current model, GOLD identifies patients needing CGA interventions by both a referral-based model and active case finding using the geriatric assessment tool developed by UK ERG and delivered by allied health professionals in a number of clinical areas (e.g., radiotherapy, chemotherapy unit and acute oncology). The GOLD clinical reviews with a multidisciplinary team are largely ‘one-stop’ face to face but with a number of other actions being managed remotely or through nurse-led telephone clinics. Patients are seen within 1–3 weeks of referral, often faster in order not to impact cancer treatment waiting times. Mostly, patients aged 70+ are not only seen but also younger patients (age 55+) as it became evident that biologically frail younger patients with poor performance status and/or multi-morbidity had similar CGA needs. A key element of this integrated service involves linking up all primary and secondary care providers. In fact, this fills a gap, where local GPs described that they tended to lose sight of older cancer patients once they have been referred to oncology. The GOLD service has proven successful by demonstrating that more older patients (aged 70+) completed chemotherapy as planned (OR 4.14, *p* = 0.006) and fewer required treatment modifications (OR 0.34, *p* = 0.006). Overall grade 3+ toxicity rates were 43.8% in those receiving GOLD service support compared to 52.9% in those receiving usual care [21].* Moreover,* 62.5% of oncologists reported that GOLD influenced decision-making. Of these, 67% reported that GOLD assisted in the evaluation of fitness for treatment, more often in favour of active treatment [22].

A number of new clinical pathways have been successfully embedded across areas within oncology and haemato-oncology via quality improvement projects. One example is a pathway with prostate cancer nurse specialist-led clinics, whereby all patients over the age of 70 years on antiandrogen therapy are referred for vascular, bone health and comorbidity risk assessment [23]. Finally, in regard to the inpatient setting, *the team piloted a model with daily GOLD clinical nurse specialist visits and clinical fellow rounds* demonstrating a significant reduction in the average length of stay.

Other centres, such as the University College London Hospitals NHS Foundation Trust (UCLH), have also been developing services for older cancer patients particularly as a part of the SCFN led by NHS Elect in England since late 2018. The focus at UCLH has been mostly on lung cancer patients admitted to oncology wards, where these have been screened by the oncology medical team using the Rockwood CFS. This triggers a referral to the Geriatric Team for a CGA which is then discussed at a newly piloted frailty multidisciplinary team meeting.

#### Manchester

The Christie NHS Foundation Trust is a standalone cancer centre without a geriatric department. However, the Oncology of Later Life Group was set-up in 2015 as a multi-professional group of individuals aiming to support and coordinate the project in geriatric oncology. The initial focus was to build awareness locally, followed by the development of an annual Onco-Geriatric Study Day since 2018. Currently, a business case is underway to develop a frailty team for a multilevel approach. The key data to support this business case were derived from the SCFN quality improvement project as the lung team joined this project led by NHS Elect in 2018. The Rockwood CFS was incorporated as an electronic tool within the standard outpatient assessment for all new lung cancer patients. Over 1000 patients have been screened since then with about 40% being vulnerable or frail (CFS 4+). Frailty correlated with ageing (*p* < 0.01) and a worse ECOG PS (*p* < 0.01) [24]. These patients were also 20%–30% less likely to be offered systemic anticancer treatment and less likely to continue beyond one cycle of treatment with more admissions. This highlighted the need for improvement in patient optimisation, selection and support. Therefore, the use of the CFS is being expanded across the trust. The team is currently working to validate this tool for use in cancer patients within the outpatient setting since this was not how it was originally developed. The gynaecological surgery team is a part of the third wave of the SCFN, with the CFS incorporated into MDT referrals and pre-operative assessments.

Meanwhile, the first dedicated oncogeriatric clinic in Greater Manchester was started in October 2018 at Wythenshawe Hospital (Manchester University NHS Foundation Trust). This focused on lung cancer patients, and all new cases aged 65+ are screened by healthcare assistants using the geriatric 8 (G8) tool. Those who fail screening are booked for a CGA, but direct referrals are also accepted. The clinic is geriatrician led, but patients benefit from subsequent referrals to allied health professionals for targeted interventions as needed. The ultimate aim is to create a multidisciplinary team. The integration of a new service is often a slow process, and whilst less than 100 patients were assessed, the number of patients completing optimal adjuvant chemotherapy saw a 15% increase since this service was implemented.

In parallel, the ambition to improve cancer outcomes in Greater Manchester has led to the development of Prehab4Cancer. It was launched in April 2019 and is the first system-wide prehabilitation and rehabilitation service for cancer patients in the UK. It focuses on optimising physical fitness and strength, nutrition and psychological well-being. Cancer patients undertake community-based prehabilitation and recovery close to their residential address, accessing the service in one of the 86 public leisure facilities overseen by Greater Manchester Active. Over 800 patients undergoing major surgical resection for colorectal, oesophagogastric and lung cancers have participated in Prehab4Cancer in its first year as a standard of care. 65% of participants were aged 70+, and the results showed that it is most useful in older patients [25]. This programme is now planned to be extended to non-surgical cancer patients as well.

In regard to research, in the medical oncology field, the ELDERS study was developed at Christie focusing on the role of immunotherapy with checkpoint inhibitors in older patients with advanced cancer. This single site, prospective cohort study enrolled 140 patients with advanced lung cancer or melanoma. The primary endpoint is safety (incidence of moderate–severe immune-related adverse events), whereas the quality of life is the key secondary endpoint. This is the first study in the field of immunotherapy to focus on older cancer patients whilst also incorporating frailty and geriatric assessments. The final results are expected to be published in 2020. Moreover, in the surgical field, the Geriatric Oncology Surgical Assessment and Functional rEcovery after Surgery trial is a real-life observational study that defined quality of life and functional recovery as its primary outcomes [26]. The hepatobiliary and pancreatic cancer unit at Manchester Royal Infirmary (Manchester University Hospitals NHS Foundation Trust) was the only centre in the UK recruiting patients to this large international trial, where a total of 1003 patients aged 70+ were recruited. The final results are also expected in 2020.

#### Newcastle upon Tyne

Newcastle upon Tyne Hospitals NHS Foundation Trust performed several practice audits, which confirmed that across a number of different tumour types, older patients were at higher risk of toxicity and have lower rates of active treatment, particularly chemotherapy [27–29].

In 2018, the lung team also joined the SCFN led by NHS Elect and implemented the Rockwood CFS early on in the cancer pathway to all those presenting with potential lung cancer [30]. Whilst not having a dedicated team for older cancer patients, similarly to the other sites within this network, the implementation of this tool was considered useful to the team and generated more in-depth discussions. Targeted interventions were then performed by an occupational therapist on site. Pilot for early assessment in lung cancer outpatient clinics is ongoing whilst experience in an outpatient clinical trial unit showed a high level of demand with significant potential for early intervention [31]. The implementation of the Rockwood CFS has been extended to other tumour types and to those admitted to oncology wards.

Research in oncogeriatrics is facilitated by the hospital’s close links with the Newcastle University Institute of Ageing and the NIHR Newcastle Biomedical Research Centre. The Newcastle 85+ study is a cohort study of people in the community aged 85+, who do not necessarily have a diagnosis of cancer. In this cohort, a series of blood tests have been shown to predict the frailty and subsequent mortality [32]. The pilot work then showed key differences in these blood tests in patients with lung cancer from the Newcastle 85+ cohort and showed that they can predict mortality [33]. In addition, sarcopenia is a key element of frailty, and this was investigated in Newcastle by assessing grip strength which when adjusted for age and gender has a prognostic value in cancer patients awaiting clinical trials [34]. Sarcopenia can also be assessed by muscle bulk on CT scan, and the radiology team developed a simple methodology to minimise intra- and interobserver variability that can be used both in clinical trials and routine practice [35].

#### Nottingham

The Nottingham University Hospitals NHS Trust established the ‘SCOPES oncology’ service (Systematic Care for Older People in Elective Surgery) in 2013. This is based on a model originally set up for older people undergoing elective orthopaedic procedures. The concept was extended to patients with upper gastrointestinal cancers who are being considered for curative surgery. In this setting, older patients (no age criteria but mostly 70+) and/or those with complex comorbidities can be referred for a geriatrician-led CGA, performed with the support of an upper gastrointestinal specialist nurse and the dietetics service. Ultimately, this team performs a fitness assessment and delivers interventions to optimise patients before surgery, promoting a timely post-operative recovery. More recently, this team has also been running a clinic for older patients suspected to have a malignancy, who require further investigations. This provides an opportunity to assess fitness for cancer treatments, to discuss the risk/benefit of further invasive investigations and to intervene/optimise patients in preparation for potential treatments. Moreover, the team is setting up a pilot for a prehabilitation service specific for lung and hopefully upper gastrointestinal cancer patients who are about to undergo treatment. The plan is that this will incorporate physical activity, nutrition, smoking cessation and psychological support. Similar to other regions in the UK, the aim is to use community sports facilities to make the service more local to patients. In August 2019, a clinical fellowship post in geriatric oncology was established with the aim to nurture the role of geriatricians embedded in an oncology department.

The University of Nottingham is also actively involved in the field and leads a unique programme on primary breast cancer in older women. The research programme has the overarching goal of optimising the management of primary breast cancer in older women and consists of three themes including the areas of breast cancer biology, geriatric assessment and quality of life and health economics. The programme offers opportunities to pursue a PhD in the field, and the team has presented its work at numerous national and international conferences, in particular, at the annual SIOG meeting. Leading on from this, the region is host to the ‘Symposium on Primary Breast Cancer in Older Women’. This is a biennial event running since 2010 organised by the University of Nottingham, in association with SIOG.

#### Sheffield

The Sheffield Teaching Hospitals NHS Foundation Trust has also been actively involved in the SCFN, led by NHS Elect in England since late 2018. The Rockwood CFS was initially implemented in 116 lung cancer patients within chemotherapy clinics. In this cohort, age was not associated with CFS; 43% of patients were classed as CFS 4–6, whereas only one patient was CFS 7–9 probably because very frail patients are not usually referred for chemotherapy; those with a CFS 4+ had longer admissions to hospital and were more likely to die within 30 days of chemotherapy. Subsequently, CFS was measured in a cohort of 103 new lung cancer patients referred to the oncology clinic. In this cohort, CFS correlated strongly with ECOG performance status; CFS 4–6 patients were more likely to stop subsequent chemotherapy after just 1 cycle, and treatment was more likely to be complicated by admission to hospital. Following this, CFS assessment was introduced earlier in the patient pathway, measured at diagnosis and recorded as a part of MDT minutes. During this process, the team further engaged with geriatric services available in the hospital. At present, a protocol for a prospective cohort of lung cancer is currently in development to define the patient groups most at risk and, therefore, most in need of early intervention. This will include several systematic health and frailty assessments. The next steps include plans to develop an oncogeriatric clinic for pre-frail and frail lung cancer patients.

The University of Sheffield coordinated the multicentre bridging of the age gap study which aimed to optimise the management of older women with operable breast cancer, reducing the age-related gap in cancer outcomes seen between older and younger women. This study collected data on over 3,400 older women (70+) with operable breast cancer from 57 sites across the UK. Recruitment was completed in June 2018, and an analysis is ongoing. The study collected real-world data on the measures of comorbidity, quality of life and functional status/frailty, in addition to treatment and outcome data (survival and quality-of-life) to identify potential areas for improvements in practice. The study also designed and developed decision support tools for clinicians and patients based on cancer registry data [36], which are freely available online [37]. The risk and outcome prediction data are stratified by patient and tumour characteristics to aid in treatment decision-making by providing more personalised outputs. These were assessed via a clustered randomised controlled trial nested within the main study.

The University of Sheffield has also recently launched a new Institute, the Healthy Lifespan Institute in 2019. This aims to bring together researchers from across the University to develop cross-disciplinary research across the range of age and multimorbidity disciplines and has a research focused on the biology and genetics of ageing, psychology of ageing, big data and machine learning to handle large datasets to better understand aspects of ageing and links with engineering to understand how to measure frailty and its treatments.

### Scotland

The NHS Scotland is comprised of 14 territorial health boards, which serves as a population of approximately 5.4 million people. Cancer care is divided into three geographical cancer networks (west, southeast and northeast) delivering cancer care in conjunction with local cancer teams.

#### West of Scotland

The Beatson West of Scotland Cancer Centre is the largest cancer centre in Scotland, and it serves 60% of the Scottish population. It hosts cancer for older persons’ service, which is the only dedicated geriatric oncology service within the West of Scotland. The service was first established in 2016 with MacMillan funding and led by two consultant geriatricians supported by a full-time elderly care haematology liaison nurse specialist. More recently, in 2019, the service was expanded for older patients with solid tumours with the appointment of an elderly care oncology liaison nurse specialist. This service is multidisciplinary and is comprised of geriatricians, oncologists, physiotherapists, occupational therapists, palliative and pastoral care. Patients aged 65+ with a diagnosis of cancer requiring specialist expertise may be referred. This includes patients requiring expertise in the management of comorbidities and polypharmacy, impaired mobility and falls, cognitive impairment, functional difficulties impairing activities of daily living, incontinence and complex discharge planning. This service has evolved predominantly within the inpatient setting. However, it also provides outpatient review for older cancer patients with a weekly consultant geriatrician- or nurse specialist-led clinic. Moreover, a weekly MDT provides an opportunity to discuss and review more complex cases. In 2019, a total of 244 inpatients were reviewed by the service with fewer outpatient reviews. However, an internal audit of inpatients revealed that a significant proportion of older patients who satisfy the referral criteria were not referred. To address the underutilisation of this service, efforts have focussed on improving service uptake through a number of initiatives and projects: the development of a geriatric focussed educational programme, raising awareness on the benefits of early integration of specialist geriatric care and, more recently, the implementation into routine practice of an electronic frailty screening tool for patients admitted via the acute oncology assessment unit which is currently being piloted.

#### North and Southeast of Scotland

Despite the recognition of service development to meet the needs of older cancer patients in these regions, at present, there is no established service. However, the Oncology and Palliative Medicine Departments in Ninewells Hospital, Dundee, started a collaboration in 2019 to develop a pilot-enhanced supportive care clinic for patients with advanced cancer, who are due to commence anticancer treatment. This multidisciplinary clinic was co-led by a medical oncologist, and a palliative care consultant will be based around the principles of realistic medicine which has been highlighted as a priority by the Scottish Government and is planned to include a CGA with capacity and expertise for personalised targeted interventions.

### Wales

NHS Wales is comprised of seven territorial health boards, which serves as a population of approximately 3.1 million people with most of its population concentrated in the south. The Wales Cancer Network provides a single, patient-focused, clinically led organisation integrating Welsh Government, all health boards and cancer service stakeholder groups.

#### North Wales

The Betsi Cadwaladr University Health Board launched in October 2019 the first pilot prehab programme aimed at cancer patients. This is delivered in a local leisure centre, and the team consists of an occupational therapist, physiotherapist and dietician, who run three sessions a week. The sessions consist of supervised exercise sessions, respiratory muscle training, diet education and anxiety management. It is aimed at patients undergoing surgery for upper-GI and colorectal cancer.

#### Southeast of Wales

In the University Hospital of Llandough in Cardiff, the first oncogeriatric service was set-up in 2017. In order to implement this service in breast cancer, a core team comprised of a breast surgeon consultant, a geriatric trainee and two breast cancer nurses, attended a leadership in improvement programme. It was felt appropriate that breast cancer was the first cancer group to develop this service as it is a neater model, with less emergencies unlike some of the other cancer groups. The Edmonton Frailty Score is used for all patients aged 70+, and this is used quite early on the pathway since it is used for all patients with a potential diagnosis of breast cancer at their initial one-stop assessment clinic. The geriatrician also attends the weekly breast multidisciplinary team meeting, where the frailty score is available. After the breast cancer diagnosis is confirmed, if the patient was screened as frail (defined as a score > 6), then an appointment is made with the geriatrician, and the patient undergoes a CGA. The implementation of this service has changed not only the assessment but also the management of older breast cancer patients in the region. Moreover, the team is currently collaborating with the primary care to link this service with a prehabilitation programme.

#### Southwest of Wales

In the Southwest Wales Cancer Centre in Swansea, the Health Education and Improvement Wales funded a Clinical Leadership Fellow in 2019 to spend a year out of programme to investigate the unmet needs of older cancer patients. The post was filled by a geriatric medicine registrar who aimed at reviewing service models and local frailty services to see how best to serve this population. Under the auspices of this fellowship, a single geriatrician-led pilot was in place between January and March 2020 which focused on patients with upper gastrointestinal cancer of any age, with comorbidities and/or polypharmacy. Patients underwent a systems review, medication review, assessment of fall risk and bone health and cognitive assessment. Data from this pilot will be used to support a business case for the continuation of this service hopefully integrated with other local services for older people.

Prehabilitation programmes are also being widely developed in the region. A study performed in South Wales demonstrated that cancer patients are more likely to have conditions requiring intervention and optimisation at the point of referral from primary care [38]. Therefore, patients with suspected lung cancer are now being offered prehabilitation classes led by physiotherapists in secondary and tertiary care centres. As many patients live in rural communities far from teaching hospitals, classes continue locally after an initial assessment. Similar group classes are being developed for older gynaecological cancer patients, with a focus on continence, exercise tolerance and sexual function.

### Northern Ireland

Health and Social Care in Northern Ireland is the designation of the service that provides care for this population of 1.9 million, about 2.8% of the whole UK population [39]. Unfortunately, between January 2017 and January 2020, there was a collapse of the devolved administration in Northern Ireland, with no responsible minister. This had a significant impact on the health service, which has been managing for several continuous years on a day-to-day basis. Within oncology, the 62-day ministerial target, whereby a cancer patient must begin treatment within 62 days of referral for suspected cancer, has not been met in over 3 years by any health trust, with the most recent figure being 48% for September 2019 [40].

The Northern Ireland Cancer Network aims to deliver improvements in outcomes, enhance experience and deliver excellence within cancer care in the region. Unfortunately, in this context, there are no projects or a healthcare strategy specific for the geriatric oncology population. Moreover, within five health trusts in Northern Ireland, there are no specific projects or guidance about the older patient with cancer. Similarly, in the research sphere, there are no dedicated regional projects either. However, in conjunction with a local cancer charity, the Belfast Health and Social Care Trust has launched a pilot of a patient-reported outcomes app called Noona. This app can be used for audit and data purposes, which may provide a useful insight about how older cancer patients are being treated and their associated toxicities. A downfall is that this app is limited to those on treatment with systemic anticancer therapy in the larger cancer centre within the region, therefore not providing any information about those not suitable for treatment as well as those having options such as surgery or radiotherapy.

## Conclusion

The care of older patients is a significant part of daily practice in oncology across the UK. However, in the vast majority of centres, the routine care of these patients does not yet include a formal multidisciplinary geriatric or frailty assessment/management. Similarly, the use of treatment toxicity prediction tools to help guide treatment decisions is not standard practice. Even where there are specialist services that care for older patients, integration with oncology is usually limited. Despite all these observations, there is a widespread acceptance that more specialised and multidisciplinary support is required for this patient population. It is, therefore, encouraging to see that over the past 5 years, geriatric oncology is now being considered within the UK healthcare strategies, and new services and collaborative projects are growing sustainably across most regions. The models of care are still very heterogeneous and adapted to the local priorities, needs and resources available. Establishing the links between oncology services and services in the primary care/community or even secondary care is crucial. Hopefully, the next 5 years will show further developments as the teams and trusts across the UK witness, acknowledge and share the benefits of dedicated oncogeriatric practice.

## Conflicts of interest

The authors declare that they have no conflicts of interest.

## Funding

The authors received no financial support for the authorship and/or publication of this article.
